# Public health round-up

**DOI:** 10.2471/BLT.23.010723

**Published:** 2023-07-01

**Ukraine’s health care under attack**

A health worker walks through a temporary ward in a hospital relocated to a bomb shelter in Kharkiv, one of the areas most affected by attacks on health care in Ukraine. According to the World Health Organization (WHO), as of 30 May there had been 1004 verified attacks on health care in the country since the Russian Federation invaded in February 2022 — the highest number ever recorded by WHO in any humanitarian emergency.

**Figure:**
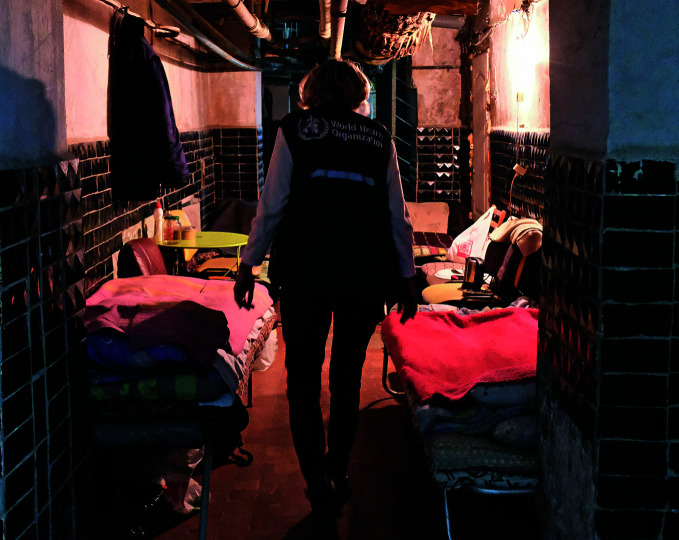
WHO/Anne Pellichero

## Sudan Emergency

The deteriorating security situation in Sudan is making the delivery of health care increasingly challenging. 

According to a situation report published by the World Health Organization (WHO) on 14 June, in Khartoum fewer than one fifth of health facilities were fully functional, while insecurity was preventing patients and health workers from reaching hospitals and health facilities were being attacked. 

Between 15 April and 8 June, 46 attacks on health care had been verified by WHO, resulting in 8 deaths and 18 injuries. 

At the time of going to press, there were also reports of military occupying the Federal Ministry of Health’s National Medical Supply Funds warehouses, where medicines for the entire country, including malaria medicines, are kept and where the national pharmacy for chronic diseases is located. The National Public Health Laboratory was also reported to be occupied.

Overall, the greatest public health risks remain the ongoing violence resulting in serious injuries, major disruptions to health care and repeated attacks on the health system. Poor access to clean water, sanitation and food, increasing the risk of malnutrition and water- and vector-borne diseases, is also a major concern.

https://bit.ly/3PeebBZ

## End of Marburg outbreaks

Health authorities in Equatorial Guinea and the United Republic of Tanzania declared the end of the Marburg virus disease outbreaks first reported by the countries in February and March 2023, respectively.

The Ministry of Health of Equatorial Guinea declared the end of the Marburg virus disease outbreak on 8 June. A total of 17 confirmed and 23 probable cases were reported from five districts in four provinces. Thirty-five of the people infected died (12 of the 17 confirmed cases and all of the probable cases).

The Ministry of Health of the United Republic of Tanzania declared the end of its first documented outbreak of Marburg virus disease on 2 June. A total of nine people were reported to have been infected (eight of the infections laboratory-confirmed), six of whom died.

WHO credited timely intervention by local authorities supported by WHO and partners with preventing the outbreaks from spreading, and for bringing them to a rapid close.

https://bit.ly/3IVRBtN

https://bit.ly/3JxJ4Ob

## Attacking health care in Ukraine

WHO reported that there had been 1004 verified attacks on health care in Ukraine since the Russian Federation invaded the country in February 2022 — the most to be recorded by WHO in any humanitarian emergency.

According to a WHO statement released on 30 May, the attacks claimed at least 101 lives, including those of health workers and patients, and injured many more. The attacks also took a heavy toll on health facilities, equipment and transport – including ambulances. WHO continues to monitor and respond to health needs in the regions most affected by the fighting.

The situation was aggravated by the destruction of the Kakhovka Dam on 6 June, responsibility for which had still to be established as of 14 June. The incident caused severe flooding, infrastructure and environmental damage, and forced communities to relocate across a wide area.

In an 8 June statement, WHO Director-General Tedros Adhanom Ghebreyesus spoke of the severe impact on the region’s water supply, sanitation systems and public health services, and said WHO was providing support across a range of response activities, including preventive measures against waterborne diseases and disease surveillance.

https://bit.ly/444p1ib

https://bit.ly/43QyhWR

## Historic World Health Assembly

WHO’s Seventy-sixth World Health Assembly ended on 30 May, and marked the Organization’s 75th anniversary.

The Assembly tackled multiple issues, ranging from best buys for noncommunicable diseases to the need for an international pandemic accord and amendments to the International Health Regulations. The Assembly also passed resolutions on an array of health topics, including the first on drowning prevention, and the health of Indigenous Peoples.

Member States approved the WHO Programme Budget for 2024-2025, which includes a 20% increase in assessed contributions, and strongly supported a proposal to prepare for the first “Investment Round” in WHO’s history, which is to be held in the last quarter of 2024 and aims to generate increased flexible and voluntary funding.

In his closing remarks, the Director-General noted the historic nature of the Assembly, and spoke of the ongoing negotiations on an international pandemic accord and amendments to the International Health Regulations as unprecedented opportunities to learn from the mistakes of the coronavirus disease 2019 (COVID-19) pandemic and ensure they are not repeated.

https://bit.ly/3X0ysgg

https://bit.ly/3J99IfU

## New emergency care alliance

WHO launched a global alliance committed to saving millions of lives by driving action on emergency critical and operative care services.

Announced on 31 May, The Acute Care Action Network will serve to drive strategic engagement of governments, communities, partners and other stakeholders, to ensure coordinated action in countries, with an emphasis on targeted support for the implementation of WHO tools in resource-limited settings. The announcement was made in the wake of the Seventy-sixth World Health Assembly resolution to strengthen access to such services.

https://bit.ly/3WLdku4

## WHO and Global Fund collaboration

WHO and the Global Fund to Fight AIDS, Tuberculosis and Malaria (the Global Fund) signed a revised Strategic Framework for Collaboration, designed to build stronger and more resilient health systems and maximize collaboration and impact in support of country, regional and global responses to major communicable diseases.

Signed on 8 June, the five-year framework aligns with the 2023-2028 Global Fund Strategy and the WHO General Programme of Work, which put communities at the centre of the health response and also address pandemic preparedness and challenges posed by climate change.

“In light of slowing progress towards ending the AIDS, tuberculosis and malaria epidemics, coupled with emerging health challenges, stronger collaboration between WHO and the Global Fund is needed more than ever,” said WHO Director-General, Tedros Adhanom Ghebreyesus.

https://bit.ly/3X0OUwR

## Water, waste, electricity and health care

More than 1 billion people visit health care facilities with inadequate or no water, sanitation, hygiene services each year. The situation is most dire in the least developed countries, where only 1 in 5 health-care facilities have basic sanitation services.

These are among the findings in *Water, sanitation, hygiene, waste and electricity services in health care facilities: progress on the fundamentals *– a new WHO and UNICEF report.

The report includes a summary of country progress in implementing the practical steps that were contained in the 2019 World Health Assembly resolution on water, sanitation, hygiene services in health-care facilities, drawing on data from an online tracking mechanism.

https://bit.ly/3p6jQ24

**Cover photo**

Children sitting outside a health centre in Shesh Pol village, Baharak district, Badakhshan province, Afghanistan.

**Figure:**
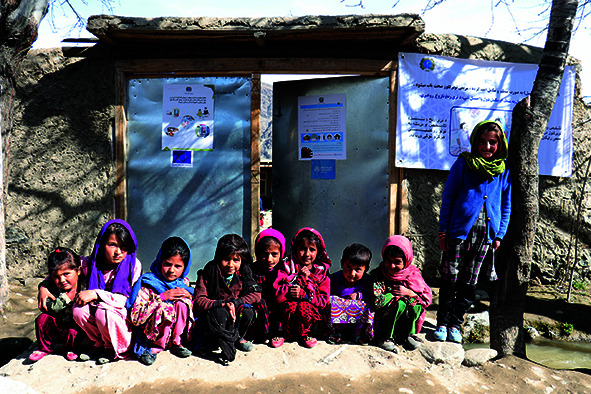
WHO/Zakarya Safari

## Drug dependence reports

WHO launched a new repository of drug dependence technical reports that will serve as an important resource for health professionals, drug policy experts and policy-makers.

Compiled by WHO’s Expert Committee on Drug Dependence and launched on 1 June, the repository comprises more than 450 psychoactive substances and is the only online, freely accessible collection of technical information regarding new psychoactive substances and medicines.

Little information is otherwise available regarding the public health risks posed by many of the substances reviewed, making the repositorty a valuable resource. Its reports will also cover therapeutic uses of psychoactive drugs.

https://bit.ly/3qo1vOs

## Let’s Move

The International Olympic Committee announced a new global initiative to inspire people to engage in daily physical activity.

Announced on 13 June, Let’s Move, which is led by Olympians and created in collaboration with WHO, will begin on Olympic Day, 23 June with an invitation for people to join them for 30 minutes in a virtual workout.

Research indicates that 1 in 4 adults and over 8 in 10 young people do not meet the recommended minimum activity levels needed for optimum health.

https://bit.ly/3P6myiR

**Looking ahead**

10–11 July 2023. Third Global Rehabilitation 2030 meeting. Geneva, Switzerland. https://bit.ly/3WOJkha

27–30 July 2023. Fourth WHO Forum on Alcohol, Drugs and Addictive Behaviours. Geneva, Switzerland. https://bit.ly/3HvqtBe

17–18 August 2023. The First WHO Traditional Medicine Global Summit. Gandhinagar, Gujarat, India. https://bit.ly/3oICahK

